# Mediastinal irradiation and valvular heart disease

**DOI:** 10.1186/s40959-022-00133-2

**Published:** 2022-04-08

**Authors:** Shivaraj Patil, Srinath-Reddi Pingle, Khalid Shalaby, Agnes S. Kim

**Affiliations:** 1 Department of Internal Medicine, University of Connecticut Heath Center, Farmington, CT USA; 2 University of Connecticut School of Medicine, Farmington, CT USA; 3 Department of Medicine, Calhoun Cardiology Center, University of Connecticut School of Medicine, 263 Farmington Avenue, Farmington, CT 06030 USA

**Keywords:** Mediastinal irradiation, Valvular heart disease, Cancer, Radiation-induced heart disease

## Abstract

Anticancer therapy has the potential to cause unwanted cardiovascular side effects. Utilization of radiation therapy to treat tumors near the heart can result in radiation-induced valvular heart disease among other cardiovascular pathologies. The aim of this review is to describe the epidemiology, pathophysiology, risk prediction, non-invasive imaging modalities and management of radiation-induced valvular heart disease with a focus on pre-operative risk assessment and contemporary treatment options.

## Highlights

• Mediastinal radiation therapy improves cancer survival but increases the risk of developing radiation-induced valvular heart disease, often in combination with other radiation-induced cardiovascular pathologies, ultimately leading to increased cardiovascular morbidity and mortality.

• Radiation-induced valvular heart disease presents after a prolonged, albeit variable, latent period and poses unique clinical challenges, underscoring the need for timely monitoring by a cardio-oncologist.

• There is an elevated risk of morbidity and mortality after valvular operations in patients with prior mediastinal radiation therapy beyond what is estimated by current preoperative risk stratification models.

• Careful patient selection and management by a multidisciplinary heart team is paramount in optimizing outcomes of radiation-induced valvular heart disease. In challenging cases associated with high operative risk, transcatheter therapies may be considered.

## Introduction

Radiation therapy (RT) plays an integral role in the treatment of various thoracic malignancies. Tumors near the heart, such as left-sided breast cancer, lymphoma, lung cancer and esophageal cancer, can expose the nearby cardiac tissue to irradiation, which may be unavoidable when delivering radiation. Historically, the myocardium was believed to be resistant to the effects of RT due to the post-mitotic state of myocytes. However, in the middle of the twentieth century, evidence began to emerge of cardiac toxicity secondary to RT [1]. Substantial doses of mediastinal irradiation can potentially result in injury to any component of the heart. The spectrum of radiation-induced heart disease is wide-ranging from early manifestations such as acute pericarditis to delayed effects: vasculopathy, valvular heart disease (VHD), pericardial disease, conduction system dysfunction and pulmonary veno-occlusive disease [2].

The overall success of RT, used alone or in combination with other modalities, has resulted in large cohorts of cancer survivors. Patients treated for Hodgkin lymphoma (HL) and breast cancer are subject to late complications from chest irradiation. Among these patients, valvular dysfunction is a frequently encountered non-coronary pathology resulting in significant morbidity and mortality [3–6]. Patients with prior RT who experience cardiac manifestations often require complex high-risk surgical interventions, posing a clinical challenge [7–11]. Treatment for VHD continues to evolve with advances in transcatheter valve therapy complementing traditional surgical approaches, resulting in expansion of available treatment modalities and the potential to transform patient care.

### Literature search methods

We conducted a PubMed search using the combinations of the words “Mediastinal Radiation”, “Chest Radiation”, “Thoracic Radiation”, “Valvular Heart Disease”,” Valvular Dysfunction”, “Aortic Stenosis”, “Aortic regurgitation”, “Mitral Stenosis”, “Mitral regurgitation”, “Tricuspid Stenosis”, “Tricuspid Regurgitation”, “Pulmonary Stenosis”, and “Pulmonary Regurgitation”. All identified manuscripts, including reviews and case series, were considered for inclusion in this review. We also reviewed the bibliographies of all identified manuscripts to find additional relevant publications. We limited the scope of our work to studies on humans, published in English since 1950. Authors reviewed all identified manuscripts for relevance to this review, and articles deemed relevant were included.

### Epidemiology

The reported prevalence of radiation-induced VHD is highly variable in part due to the differences in study design, baseline characteristics of various cancer survivor cohorts, heterogeneous cardiotoxic exposures, and lack of established methodologies used to characterize valve dysfunction. In a cohort of five-year childhood cancer survivors who had received mediastinal RT (MRT), the observed prevalence of one or more valvular abnormalities at the time of first echocardiographic evaluation was 43.1% at a median age of 22 years, after a median follow-up of nearly 15 years [12]. Studies among survivors of HL with a history of MRT have shown the prevalence of valvular abnormalities as detected by echocardiography to range from 2.8–61% [13, 14].

A cross-sectional study of 82 HL survivors by Bijl et al. compared valvular dysfunction among patients who received MRT with those who did not and found that left-sided valvular lesions, aortic regurgitation (38.2%) followed by mitral regurgitation (36.7%), were the most common abnormalities [14]. Left-sided valvular pathologies are generally more common in the general population, likely due to the higher-pressure system on the left side of the heart as compared with the right side and may explain why the mitral and aortic valves are more commonly affected than pulmonary and tricuspid valves [15, 16]. Tricuspid regurgitation (20.4%) was the most common right-sided valvular lesion. Aortic stenosis (AS) was the most common obstructive valvular dysfunction followed by mitral stenosis. In contrast, a study by Cella et al. reported a higher occurrence of mitral (25.0%) and tricuspid (14.3%) compared with aortic (5.4%) valve disease among HL survivors. This discrepancy is possibly due to the relatively shorter follow-up period at the time of echocardiographic assessment in the latter study [17].

For all types of VHD, the prevalence and severity were highest in the group of patients who had received RT more than 20 years prior to evaluation, a finding also supported by the study of nearly 300 asymptomatic survivors by Heidenreich et al., highlighting the latency in the development of valvular lesions [18]. In breast cancer survivors, the prevalence of VHD has been reported to be between 0.4–4.2% among patients exposed to RT. The risk of developing VHD is increased by approximately fifty percent in left-sided, compared with right-sided, breast cancer, highlighting the implication of mean heart dose in the development of radiation-induced toxicity [5, 19, 20]. In a large, population-based cohort comprising 70,230 surgically treated breast cancer patients receiving adjuvant RT, death due to VHD was more frequent (standardized mortality ratio 1.28, 95% CI 1.08–1.52) compared with the general population [21].

### Risk factors and pathophysiology

The strongest risk factor for developing radiation-induced VHD is the total radiation dose delivered to the mediastinum, more specifically the total dose delivered to the heart valves. In a nested cohort study of 1852 patients treated for HL between 1965 and 1995, 5% had VHD (N = 89) with 74% of lesions being severe or life-threatening (*N* = 66) [22]. A dose–response relationship of progressively nonlinear increases in clinically significant VHD was noted with relative risks of 1.4, 3.1, 5.4, and 11.8 for affected heart valves receiving ≤ 30 Gy, 31 to 35 Gy, 35 to 40 Gy, and ≥ 40 Gy, respectively. With advancements in RT in the current era, the cumulative risk of developing VHD is estimated at 1.4% at 30 years among patients receiving MRT for HL with a standard radiation dose of 20 or 30 Gy [22]. The volume of heart irradiated is also a major risk factor for the development of VHD. In one analysis of patients treated between 2002 and 2008 with chemotherapy and involved-field RT, radiation to cardiac sub-volumes correlated with the occurrence of VHD. The volume of left atrium exceeding 25 Gy and the volume of left ventricle exceeding 30 Gy predicted mitral and aortic valve dysfunction [17].

A meta-analysis of 40,781 breast cancer survivors who had received modern RT showed that the risk of developing of VHD was 1.97 compared to those who did not undergo RT (95% CI: 1.07–3.67, *p* = 0.03). The mean heart dose reported was 6.3 Gy for the whole heart, which is higher than contemporary radiation doses [23, 24]. There is a higher whole heart radiation dose exposure in individuals receiving RT for left-sided breast cancer compared to right-sided breast cancer, primarily affecting the left ventricle, left anterior descending artery and right ventricle due to their anatomic proximity [25]. Techniques like prone positioning, respiratory gating, and deep inspiration breath hold displace the breast away from the heart and therefore reduce radiation burden to the heart (Table 1).

**Table 1:** Cardiac-Sparing Modalities and Techniques in Radiation Therapy

Cardiac-Sparing Technique	Description	Type of Cancer
Cardiac Displacement		
Respiratory Gating	Tracking patient’s natural respiratory motion and delivering radiation precisely when the tumor is in the treatment field and farthest from the heart	Breast, Lymphoma, Lung, Esophagus
Deep Inspiration Breath Hold (DIBH)	Radiation is administered during maximal inspiration and breath hold, when the heart is pulled away from the chest wall due to flattening of the diaphragm and expansion of the lungs	Breast, Lymphoma
Prone Positioning/ Lateral Decubitus Positioning	Radiation is delivered to the tumor with patient lying prone or lateral decubitus on a specially designed table to maximally displace the heart from the treatment field	Breast
Radiation Treatment Modality/Technique		
Involved Site Radiation Therapy (ISRT) and Involved Node Radiation Therapy (INRT)	Reduction in radiotherapy field size to involved-nodal tissue detected using modern imaging techniques (PET-CT/MRI), thus sparing surrounding uninvolved nodal and non-nodal anatomic structures	Lymphoma
Three-Dimensional Conformal Radiation Therapy (3D-CRT)	3D reconstruction of the tumor and surrounding structures using CT and/or MRI imaging data to guide radiation planning by beam placement. Radiation can be delivered from any angle; multiple radiation beams from different angles can be combined to deliver maximal dose to the tumor while relatively sparing normal tissue	Breast, Lymphoma, Lung, Esophagus
Intensity-Modulated Radiation Therapy (IMRT)	An advanced form of 3D-CRT that utilizes varying intensity of smaller radiation beams (beamlets) using computerized inverse planning, enabling precise delivery of radiation dose to the tumor and improving normal tissue sparing	Breast, Lymphoma, Lung, Esophagus
Volumetric-Modulated Arc Therapy (VMAT)	An extended form of IMRT, in which the radiation source is continuously rotated around the patient, allowing delivery of therapy from a full 360° beam angle, with added advantage of improved delivery of radiation dose to the target in lesser time	Breast, Lymphoma, Lung, Esophagus
Image-Guided Radiation Therapy (IGRT)	Integration of imaging prior to and during each radiation treatment, typically CT-guided and recently MRI-guided, allowing more precise localization of the tumor bed. IGRT permits significantly better sparing of normal tissue while promoting dose-escalation to the tumor when incorporated with IMRT	Breast, Lymphoma, Lung, Esophagus
Accelerated Partial Breast Irradiation	An approach that treats only the lumpectomy bed plus a 1–2 cm margin, rather than the whole breast, therefore sparing normal tissue by decreasing the target volume of radiation	Breast
Proton Beam Therapy	Proton beams have a distinct property compared to photon beams: they quickly lose energy toward the end of their range (Bragg peak), thus limiting radiation dose beyond the target	Breast, Lymphoma, Lung, Esophagus

Interestingly, there are histopathological differences noted in the affected valves with a degenerative calcific process observed in patients with breast cancer as opposed to a predominantly fibrotic process in lymphoma patients who have received RT. This difference is likely due to the young age of radiation exposure in lymphoma patients [26]. A recent observational matched cohort study of patients with moderate AS with and without prior MRT who underwent serial echocardiograms showed similar rates of AS progression regardless of the underlying primary malignancy treated with RT. Patients with prior MRT had significantly increased mortality despite undergoing aortic valve replacement sooner compared with their matched controls, highlighting the adverse impact of radiation even after the correction of underlying valve abnormality [27].

Myocardial fibrosis and vasculopathy due to the direct toxic effects of radiation result in ventricular remodeling, which in turn can increase the risk of developing valvular dysfunction. A few studies have reported an increased risk of VHD with concomitant exposure to anthracycline therapy in a dose-dependent manner [4, 28, 29]. However, Cutter et al. did not find a significant association of VHD with anthracycline exposure [22]. This may be due to the latter report involving only patients with primary VHD, while other studies included all diagnoses of valve dysfunction, including secondary valvulopathy following ischemic heart disease or cardiomyopathy.

Other risk factors for VHD include early age of exposure to RT, hypertension, smoking, and hyperlipidemia [4, 14, 18, 23, 24, 30]. The presence of congenital heart disease has also been reported to increase the risk of valvulopathy [12]. Interestingly, obesity at the time of HL diagnosis and splenectomy, although no longer used for the treatment of HL, have also been associated with an increased risk of radiation-induced valve damage. The relationship between splenectomy and valve disease is not well understood and may represent a correlation rather than causation [4, 31].

Although the precise pathophysiologic mechanisms of radiation-induced valvulopathy are not completely understood, irradiation is thought to have a direct effect on the pathologic fibrosis and calcification of the valvular apparatus. Due to the avascular nature of valve tissue, the mechanism of injury is believed to be distinct from radiation-induced damage to the myocardium and vasculature. There is a lack of histological markers of chronic inflammation or neovascularization on tissue specimens [26, 32]. Fibrotic changes in the vasculature are mediated via activation of inflammatory cascade through radiation-induced endothelial cell injury [33]. In contrast, injury to the valve interstitial cells (VICs) has been implicated in the pathogenesis of valve damage from radiation. VICs originate from valve endothelial cells during embryogenesis via migration into the underlying matrix and undergoing endothelial‐to‐mesenchymal transformation. Pathological activation of VICs leads to their differentiation into myofibroblasts and osteoblasts via cytokines that act in an autocrine and paracrine fashion. In addition, mechanical stress contributes to this transformation, explaining the higher incidence of left-sided lesions compared with right-sided lesions. These cells further produce collagen, extracellular matrix, and osteogenic factors, such as bone morphogenic protein 2, transcription factor Runx2, osteopontin and ALP, all of which play a role in creating a milieu for abnormal calcium deposition in irradiated valves [34, 35].

### Clinical prediction model for radiation-induced valvular heart disease

Use of RT has significantly improved overall and disease-free survival for patients with cancer, albeit with a risk of increased morbidity because of injury to surrounding normal tissue. To keep the tissue toxicity at an acceptable level, radiobiological models such as normal tissue complication probability (NTCP) have been developed to estimate the risk of radiotoxicity to normal tissue. These models are utilized in treatment planning to minimize adverse effects from irradiation. NTCP models, such as the Lyman-Kutcher-Burman (LKB) and Relative Seriality (RS) models, are the most well-known and traditionally accepted methods for predicting toxicity after radiation treatment. However, these traditional models have inadequately predicted the risk of radiation-induced VHD based on conventional heart dose-volume histograms alone due to lack of substructure contouring. Improved statistical machine learning methods like “Least Absolute Shrinkage and Selection Operator” (LASSO) have been adopted to build multivariate NTCP models to improve risk prediction, however, these models need further validation [36].

### Advances in radiation therapy

Growing recognition of the adverse cardiac effects of mediastinal radiation and technological advances in the field of radiation therapy have led to the development of cardiac-sparing modalities and techniques. In contrast to the 1970s and 1980s, modern radiation therapy employs multiple techniques to minimize the mean heart dose, such as deep inspiration breath hold, intensity-modulated radiation therapy, and proton beam therapy (Table 1) [37–42]. These advances are aimed at improving the therapeutic ratio, i.e., delivering maximal effective radiation to the tumor while minimizing the risk of radiation toxicity to surrounding healthy tissue [37–39]. Furthermore, employing imaging techniques, such as ECG-gated CT coronary angiography and/or MRI, can facilitate incorporation of cardiac substructures (i.e., valves, coronaries, cardiac chambers) into radiation treatment planning and delivery, allowing better estimation of radiation dose to specific substructures of the heart and mitigation of cardiac injury [40–42]. The selection of techniques and modalities is a complex process and influenced by several factors, particularly the availability of technology, patient anatomy, and target volumes. An interdisciplinary discussion involving the oncologist, radiation oncologist and cardio-oncologist is key to determine optimal radiation therapy in improving patient survival, especially in patients with established cardiovascular disease or risk factors [43, 44]. The impact of modern cardiac-sparing radiation therapy on VHD is unknown, largely due to its delayed manifestation, and is an important area for future research.

### Multi-modality non-invasive imaging

**Fig. 1:**
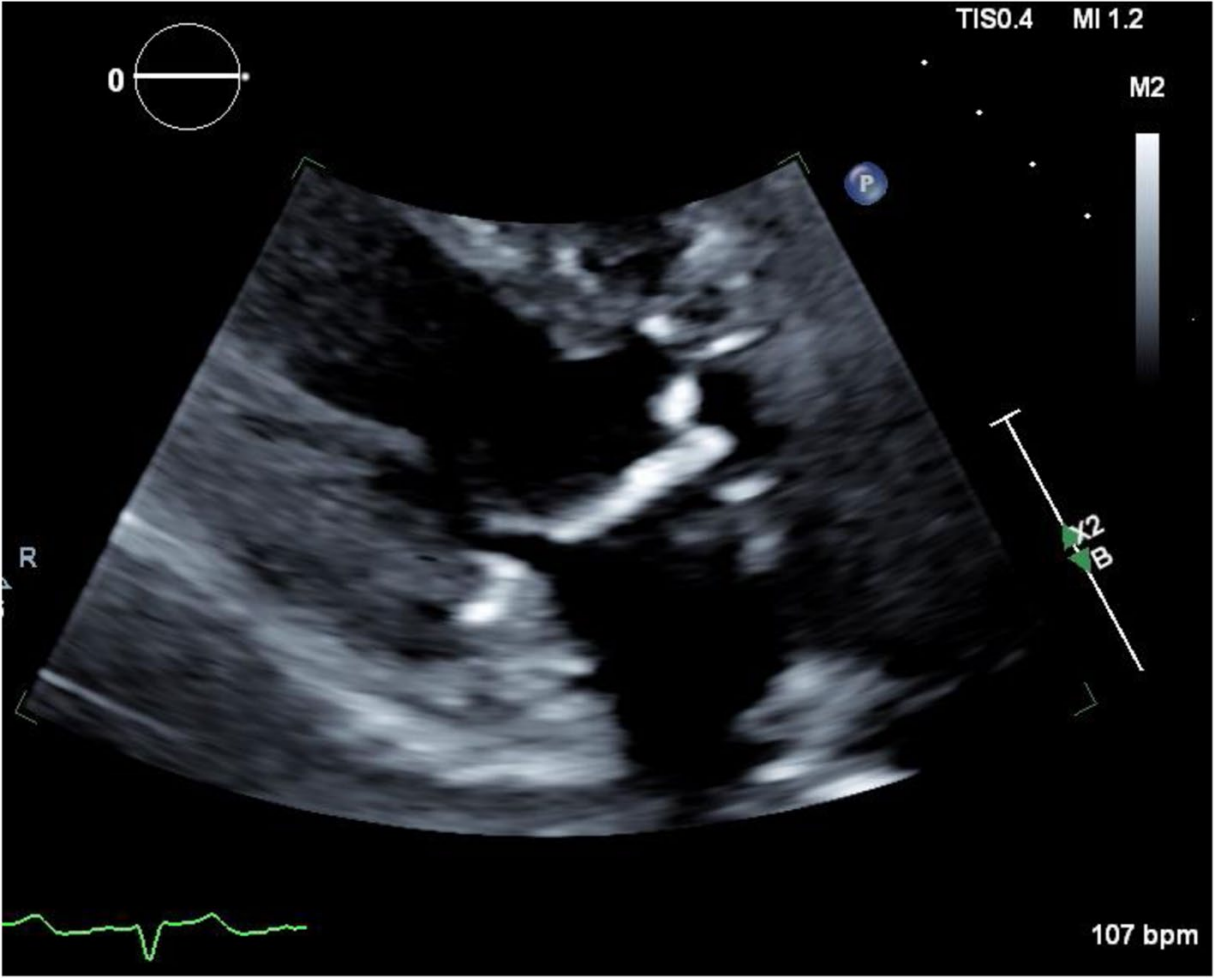
Transthoracic echocardiography measuring aorto-mitral curtain thickness. Example TTE image of aorto-mitral curtain thickness in a 55-year-old man with a history of mediastinal radiation therapy for non-Hodgkin lymphoma at the age of 30. He underwent aortic and mitral valve replacement for symptomatic severe valvular stenosis

Transthoracic echocardiography is the first-line imaging modality for detecting VHD (Table 2). Echocardiographic abnormalities include diffuse valve thickening due to fibrosis and focal or contiguous calcification in the valvular apparatus resulting in restricted motion of leaflets, initially causing regurgitation with eventual progression to stenosis. Left-sided valves are more often affected than right-sided valves. The aortic root, aortic valve annulus, aortic valve leaflets, aortic-mitral inter-valvular fibrosa, mitral valve annulus, and the base and mid portions of the mitral valve leaflets are typically affected with sparing of mitral valve tips and commissures [45]. Special focus should be given to the measurement of aorto-mitral curtain (AMC) thickness due to its prognostic importance (Fig. 1). An AMC thickness of greater than 6 mm has been shown to be independently associated with increased mortality [46]. The severity of valvular dysfunction should be graded based on the guidelines from the European Association of Cardiovascular Imaging and the American Society of Echocardiography. In addition, echocardiography can provide valuable information regarding ventricular systolic and diastolic dysfunction as well as pericardial pathology, importantly constrictive pericarditis.

**Table 2:** Common Echocardiographic Findings of Radiation-induced Valvular Heart Disease

1	Diffuse valve thickening due to fibrosis
2	Focal or contiguous calcification of valvular apparatus with restricted motion of leaflets
3	Initial regurgitation with eventual progression to stenosis
4	Fibrosis/calcification of aortic root, aortic valve annulus, aortic valve leaflets, aortic-mitral inter-valvular fibrosa, mitral valve annulus, and the base and mid portions of the mitral valve leaflets with typical sparing of mitral valve tips and commissures
5	Aorto-mitral curtain (AMC) thickness
6	Ventricular systolic and diastolic dysfunction
7	Pericardial pathology, importantly constrictive pericarditis

Multidetector cardiac computed tomography (MDCT) and cardiac magnetic resonance (CMR) are useful adjunct imaging modalities. They provide complimentary information for pre-procedural planning, including assessment of annular shape and size for aortic, mitral, and tricuspid valves, and ileo-femoral vasculature. Presence of significant calcification of the valvular apparatus may preclude valvular repair. Extreme radiation-induced calcification of the ascending aorta (“porcelain aorta”) can increase the perioperative risk of embolic stroke due to its manipulation. Therefore, its detection is important to determine the suitability for aortic cross-clamping and cannulation in patients undergoing cardiac surgery. For patients undergoing redo surgeries, MDCT provides critical information regarding anatomical relationship of various cardiovascular structures to the sternum and the extent of mediastinal fibrosis, which may preclude the use of open cardiac surgery and favor minimally invasive techniques for treatment [47]. Among individuals with inadequate information by transthoracic or transesophageal echocardiography, CMR can provide both structural and functional data regarding valve dysfunction, as well as information on ventricular function, mass, volume, regional wall motion abnormalities, and pericardial thickening, effusions, and features of constrictive physiology [48]. Coronary vasculopathy is common in patients with radiation-induced VHD and commonly affects the ostia or proximal coronary arteries. This can be detected non-invasively by stress echocardiography, stress CMR, coronary CTA, radionuclide myocardial perfusion imaging with single photon emission CT (SPECT), or positron emission tomography (PET).

### Management

Annual follow-up with a cardiologist or cardio-oncologist for history and physical examination and utilization of multimodality cardiac imaging when appropriate are invaluable for the early detection and timely intervention of radiation-induced VHD (Table 3). Development of new cardiopulmonary symptoms or physical exam findings, particularly murmur, should prompt immediate diagnostic evaluation with echocardiography regardless of the time from MRT. The risk factors for developing VHD include anterior or left-sided chest wall irradiation, exposure to a high cumulative dose of radiation (> 30 Gy) or a high daily fraction of radiation > 2 Gy, lack of shielding, young age at RT (< 50 years), concomitant chemotherapy, presence of pre-existing cardiovascular disease, or presence of cardiovascular risk factors (diabetes mellitus, hypertension, hyperlipidemia, obesity, and smoking). Apart from these traditionally described risk factors, there is growing evidence to include the radiation dose distribution to cardiac substructures to better identify high-risk individuals [49]. An asymptomatic individual is considered high risk if a cardiac structure was in the radiation field, typically observed in anterior or left-sided chest irradiation, with at least one other traditional risk factor for radiation-induced heart disease.

**Table 3:** Management Recommendations for the Prevention and Detection of Radiation-induced Valvular Heart Disease

1	Annual follow-up with a cardiologist or cardio-oncologist for history and physical examination
2	Assess risk factors for developing VHD: anterior or left-sided chest wall irradiation, exposure to a high cumulative dose of radiation (> 30 Gy) or a high daily fraction of radiation > 2 Gy, lack of shielding, young age at radiotherapy (< 50 years), concomitant chemotherapy, presence of pre-existing cardiovascular disease, or presence of cardiovascular risk factors (diabetes mellitus, hypertension, hyperlipidemia, obesity, and smoking)
3	For individuals with symptoms or murmur, check echocardiography
4	For low-risk asymptomatic individuals, screening for VHD with echocardiography is recommended at 10 years
5	For high-risk asymptomatic individuals, surveillance imaging for VHD should begin sooner, typically at 5 years
6	Asymptomatic individuals should undergo surveillance imaging every 5 years if initial screening echo is normal
7	Optimal management of underlying cardiovascular risk factors (hypertension, diabetes, hyperlipidemia, smoking, obesity, sedentary lifestyle, obstructive sleep apnea) is imperative

For low-risk asymptomatic individuals, screening for VHD with echocardiography is recommended at 10 years. For high-risk asymptomatic individuals, surveillance imaging for VHD should begin sooner, typically at 5 years. Asymptomatic individuals should undergo surveillance imaging every 5 years if initial screening echocardiogram was normal [45]. More recently, the international cardio-oncology society recommends screening with echocardiography as early as 6–12 months in high-risk individuals and at least one echocardiogram within 5 years of RT in all individuals in whom the heart was in the radiation field [50]. Detection of valvular and/or ventricular dysfunction should prompt closer interval follow-up depending on the severity of the abnormality detected. Radiation-induced coronary artery disease often develops prior to significant valvular dysfunction and warrants earlier detection, typically 5 years after completion of RT and repeated surveillance at 5-year intervals. Patients with abnormal stress tests or those being planned for valvular intervention should undergo left heart catheterization to assess the coronary anatomy and to confirm the findings of noninvasive stress testing. The extent and severity of underlying atherosclerotic and/or radiation-induced coronary vasculopathy may have significant implications in planning for cardiac surgery [45, 48, 50].

Optimal management of underlying cardiovascular risk factors (hypertension, diabetes, hyperlipidemia, smoking, obesity, sedentary lifestyle, obstructive sleep apnea) is imperative in these patients. Radiation-induced VHD progresses over time and ultimately will require invasive structural interventions to relieve significant lesions. Patients with exposure to MRT can develop complex cardiac disease involving valvular, coronary, ventricular, and conduction system abnormalities of the heart as well as simultaneous disease of the surrounding structures. There are no specific guidelines on the timing of intervention for patients with radiation-induced VHD. Interventions should be performed according to current existing national and international guidelines [51, 52]. However, due to the presence of multiple structural abnormalities in these patients, a delayed surgical approach with an aim to perform a complete operation at the first surgery is preferred to avoid the risk of cardiac reoperation, which is associated with significant morbidity and mortality [48, 53]. A comprehensive pre-operative evaluation should be performed including echocardiography, coronary angiography, MDCT, and pulmonary function testing.

Surgical risk stratification with current pre-operative risk stratification tools for cardiac valve surgery do not account for the adverse effects and complications related to prior MRT and may underestimate the true risk. A retrospective analysis by Wu et al. of 173 patients (mean age, 63 ± 14 years, 75% women, mean EuroSCORE 7.8 ± 3) with radiation-induced heart disease undergoing cardiac surgery matched to 305 controls based on age, gender, and type of procedure revealed a higher proportion of death in the RT group than in the comparison group (55% versus 28%; *p* < 0.001) over a mean follow-up of 7.6 ± 3 years, despite similar EuroSCOREs [10]. A retrospective analysis by Ejiofor et al. of 261 patients (mean age 62.6 ± 12.1 years; 67% women) with prior MRT who underwent valvular operations (82% primary; 18% re-operative procedures) revealed that prior RT adversely affected long-term survival compared with 836 nonradiated matches: HR 2.24 (95% CI 1.73–2.91) for primary operations and HR of 3.19 (95% CI 1.95–5.21) for redo operations. Patients with prior MRT had higher operative mortality compared with nonradiated matches: primary operation (3.8% vs. 0.8%; *p* = 0.004) and redo operation (17% vs. 2.3%; *p* = 0.001). The risk of returning to the operating room (8.5% vs. 1.9%), median time on a ventilator (20.3 h vs. 7 h), length of stay in the intensive care unit (122 h vs. 51 h), and overall hospital length of stay (15 days vs. 7 days) were significantly greater in the re-operative group compared with primary cases [53]. An analysis by Handa et al. of 60 patients with prior MRT who underwent valvular operations reported increased rates of early mortality in patients with constrictive pericarditis (40% vs. 6%, *p* = 0.011). In the same cohort, reduced preoperative ejection fraction and longer cardiopulmonary bypass times were also associated with early mortality [7].

Among 230 patients undergoing cardiac surgery after thoracic radiation, the proportion of peri-operative morbidity, in-hospital death, and long-term mortality was highest in patients who had received extensive radiation exposure compared with those who received variable and tangential radiation exposure, underscoring the fact that more extensive radiation exposure is associated with worse survival after cardiac surgery [9]. Therefore, a good understanding of the nature of radiation exposure to the heart and its substructures is prudent to accurately characterize pre-operative risk prior to cardiothoracic surgery and determine the most appropriate therapeutic plan, underscoring the need for multidisciplinary collaboration between the radiation oncologist, cardio-oncologist, cardiac surgeon, and other cardiac sub-specialists.

The development of transcatheter valve interventions provides an alternative to surgical valve interventions in intermediate and high-risk patients. Observational data is mainly available for patients with MRT undergoing transcatheter aortic valve replacement (TAVR) for AS since therapies for mitral and tricuspid valve are still evolving with limited experience in this setting. For patients with prior MRT, TAVR should be considered in those with isolated AS, high risk for surgical aortic valve replacement (SAVR), absence of advanced coronary artery disease, and without an excessive risk for coronary obstruction or annulus rupture. A retrospective study of 110 patients (55 TAVR, mean age 72 years, mean STS score 5.1%; 55 SAVR, mean age 60 years, mean STS score 1.6%) with severe AS and prior MRT compared outcomes of TAVR and SAVR with the expected mortality based on STS risk score and showed that the 30‐day all‐cause observed‐versus‐expected mortality ratio was lower in the TAVR than in the SAVR group (TAVR 0.33 [95% CI 0.01–1.86] vs. SAVR 5.00 [95% CI 1.62–11.67], *p* = 0.005). There was no difference in 30‐day or 1‐year all‐cause mortality on crude analysis; however, after adjusting for baseline STS score, 30‐day and 1-year all‐cause mortality were significantly lower in the TAVR group. On the contrary, readmissions were higher for the TAVR group primarily due to heart failure [54]. In one study, intermediate and high-risk patients (STS-PROM > 3%) undergoing isolated AVR, TAVR had improved survival compared to SAVR at 48 months. However, in low-risk patients, TAVR and SAVR were equivocal in offering survival advantage, thus allowing for a SAVR-first approach in younger patients with acceptable peri-operative risk with the possibility of a future valve-in-valve TAVR [55].

A study analyzing 610 patients (75 with prior MRT, mean STS score of 9.11%; 535 with no prior MRT, mean STS score 9.02%) with severe AS undergoing TAVR reported higher all-cause mortality (29% vs.15%, *p* < 0.01) and major adverse cardiovascular events (defined as cardiovascular mortality, stroke, acute myocardial infarction or revascularization, and heart failure) (57% vs. 27%, *p* < 0.001) in patients with prior MRT undergoing TAVR compared with the general population during a mean follow up of 17.1 months [56]. A recent meta-analysis showed similar 30-day mortality, safety, and efficacy outcomes of TAVR in patients with prior MRT compared with those without prior MRT. However, patients with prior MRT had higher 1-year mortality (OR 1.97, CI 1.15 to 3.39, *p* = 0.01) and CHF exacerbation (OR 2.03, CI 1.36 to 3.04, *p* = 0.0006) [57]. This further highlights the ongoing adverse effects of radiation on the heart despite correction of significant lesions and the need for more prospective randomized trials to identify patients with prior MRT who would benefit from percutaneous transcatheter valvular interventions. Careful selection of patients for surgical versus transcatheter interventions by a multidisciplinary heart team is necessary to optimize outcomes in patients with radiation-induced VHD.

Ultimately, cardiac transplantation may be the only therapeutic option in patients with advanced disease states not amenable to either conventional surgical or transcatheter interventions. In a small series of 12 patients with severe radiation-induced cardiomyopathy who underwent orthotopic cardiac transplantation at a single center, there was no difference in mortality between the MRT and non-MRT groups with 1-year, 5-year, and 10-year survival of 91.7%, 75%, and 46.7% compared with 92.6%, 81.8%, and 66.5%, respectively (*p* = 0.17) [58]. However, in a relatively larger series of 75 patients from United Network for Organ Sharing registry transplanted for radiation-induced cardiomyopathy, post-transplantation survival was lower in these patients than in those with other forms of cardiomyopathies and heart failure etiologies, mainly because of higher early postoperative mortality (6-month mortality was 21% for radiation-induced cardiomyopathy, 8% for other restrictive cardiomyopathies, and 9% for other cardiomyopathies) [59]. Due to prior exposure to radiation and chemotherapy, these patients are prone to developing recurrent malignancies, and the risk further increases due to post-transplantation immunosuppressive therapy. Thus, careful evaluation of these patients is mandatory to assess freedom from primary or secondary malignancies when considering transplant candidacy.

## Conclusion

**Fig. 2:**
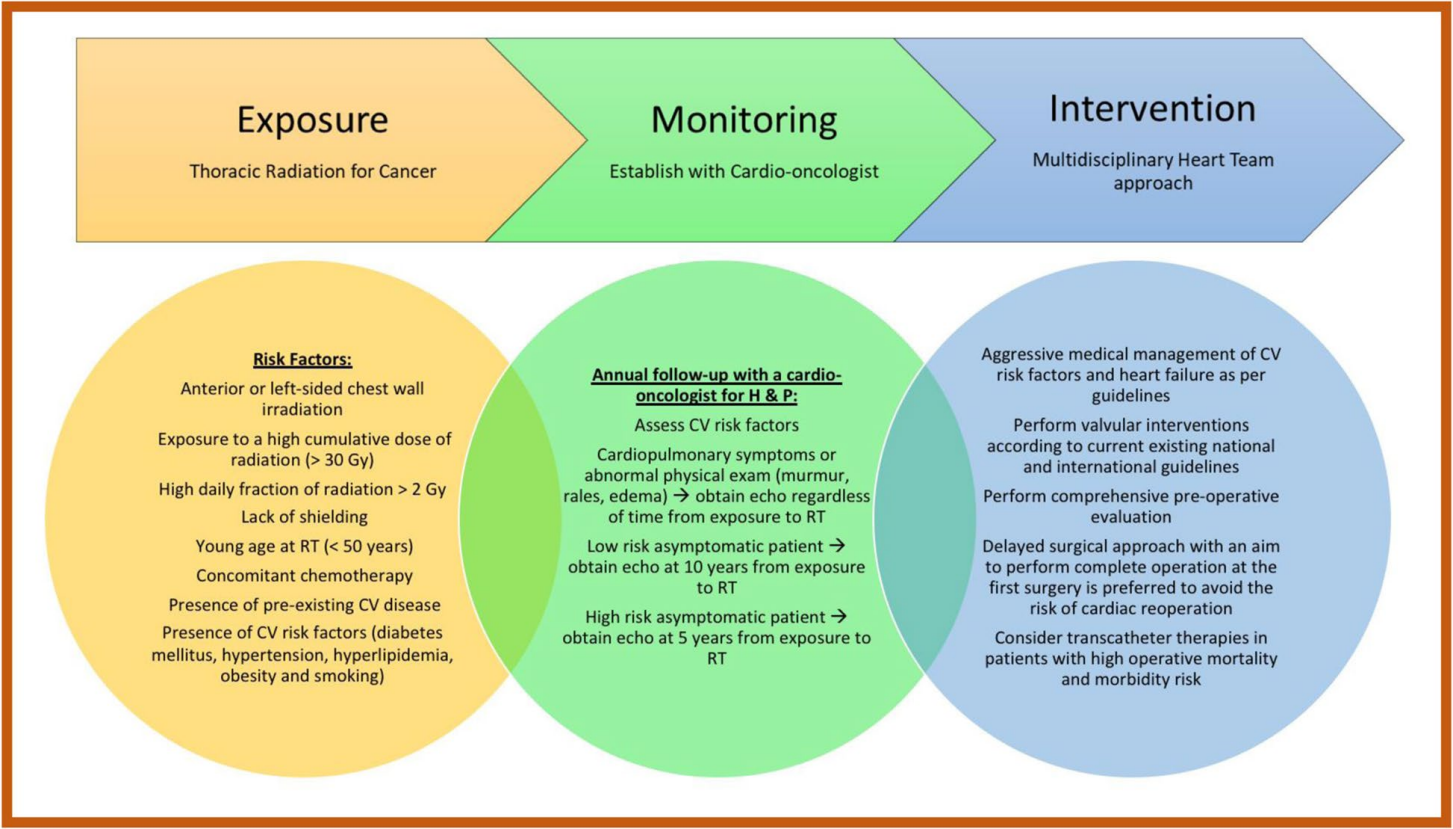
Mediastinal irradiation and valvular heart disease. An illustration of risk factors for the development of valvular heart disease (VHD) due to mediastinal radiation therapy (MRT), how to monitor patients with prior MRT, and therapeutic approaches for patients who develop VHD

Radiation-induced VHD is a major cause of cardiovascular morbidity and mortality among cancer survivors. Establishing care with a cardio-oncologist for close monitoring of heart health will help facilitate early detection and allow for timely preventative and therapeutic interventions (Fig. 2). Effects of prior mediastinal RT should be included in pre-procedural risk assessment, and decisions regarding intervention must be holistically individualized with the help of a multidisciplinary heart team. Individuals with radiation-induced VHD are generally high-risk surgical candidates, and an open surgical approach is commonly contraindicated due to underlying hostile anatomy and risk of potential complications. Those patients who undergo surgical correction of radiation-induced valvulopathy have worse outcomes compared with their traditional counterparts for similar interventions, making minimally invasive transcatheter therapy a more appealing choice. Despite advances in transcatheter technology in treating VHD, particularly AS, the efficacy and safety of percutaneous valve interventions in this specific patient population need further validation to determine optimal treatment.

## Data Availability

Not Applicable.

## Abbreviations

RT: Radiation therapy
VHD: Valvular heart disease
HL: Hodgkin lymphoma
MRT: Mediastinal radiation therapy
AS: Aortic stenosis
VICs: Valve interstitial cells
NTCP: Normal tissue complication probability
LASSO: Least Absolute Shrinkage and Selection Operator
AMC: Aorto-mitral curtain
MDCT: Multidetector cardiac computed tomography
MRI: Magnetic resonance imaging
CMR: Cardiac magnetic resonance
STS: Society of Thoracic Surgeons
TAVR: Transcatheter aortic valve replacement
SAVR: Surgical aortic valve replacement
